# Attrition one year after starting antiretroviral therapy before and after the programmatic implementation of HIV “Treat All” in Sub-Saharan Africa: a systematic review and meta-analysis

**DOI:** 10.1186/s12879-023-08551-y

**Published:** 2023-08-28

**Authors:** Richard Makurumidze, Tom Decroo, Bart K. M. Jacobs, Simbarashe Rusakaniko, Wim Van Damme, Lutgarde Lynen, Tinne Gils

**Affiliations:** 1 Institute of Tropical Medicine, Clinical Sciences Department, Antwerp, Belgium; 2 Faculty of Medicine and Health Sciences, Department of Primary Health Care Sciences, University of Zimbabwe, Harare, Zimbabwe; 3 Faculty of Medicine & Pharmacy, Gerontology, Vrije Universiteit Brussel (VUB), Brussels, Belgium; 4 Research Foundation of Flanders, Brussels, Belgium; 5 Global Health Institute, University of Antwerp, Antwerp, Belgium

**Keywords:** HIV, “Treat All, Attrition, Retention, Antiretroviral therapy, Sub-Saharan Africa, Systematic review

## Abstract

**Introduction:**

Evidence on the real-world effects of “Treat All” on attrition has not been systematically reviewed. We aimed to review existing literature to compare attrition 12 months after antiretroviral therapy (ART) initiation, before and after “Treat All” was implemented in Sub-Saharan Africa and describe predictors of attrition.

**Methods:**

We searched Embase, Google Scholar, PubMed, and Web of Science in July 2020 and created alerts up to the end of June 2023. We also searched for preprints and conference abstracts. Two co-authors screened and selected the articles. Risk of bias was assessed using the modified Newcastle–Ottawa Scale. We extracted and tabulated data on study characteristics, attrition 12 months after ART initiation, and predictors of attrition. We calculated a pooled risk ratio for attrition using random-effects meta-analysis.

**Results:**

Eight articles and one conference abstract (nine studies) out of 8179 screened records were included in the meta-analysis. The random-effects adjusted pooled risk ratio (RR) comparing attrition before and after “Treat All” 12 months after ART initiation was not significant [RR = 1.07 (95% Confidence interval (CI): 0.91–1.24)], with 92% heterogeneity (I^2^). Being a pregnant or breastfeeding woman, starting ART with advanced HIV, and starting ART within the same week were reported as risk factors for attrition both before and after “Treat All”.

**Conclusions:**

We found no significant difference in attrition before and after “Treat All” one year after ART initiation. While “Treat All” is being implemented widely, differentiated approaches to enhance retention should be prioritised for those subgroups at risk of attrition.

**PROSPERO number:**

CRD42020191582.

**Supplementary Information:**

The online version contains supplementary material available at 10.1186/s12879-023-08551-y.

## Introduction

At the end of 2020, there were an estimated 38 million people living with HIV (PLHIV) globally, and of these, 25 million (67%) were in Sub-Saharan Africa [1]. To control HIV-related mortality and HIV transmission, UNAIDS launched the 90–90-90 targets: 90% of PLHIV should know their status, 90% of people with confirmed HIV infection should receive antiretroviral therapy (ART), and 90% of those on ART should be virally suppressed by the end of 2020 [2]. The region of Eastern and Southern Africa made important progress toward these 90–90-90 targets, with an estimated 87% of PLHIV aware of their status, 72% of them receiving ART, and 65% of them having achieved viral load (VL) suppression by 2020. In Western and Central Africa, the 90–90-90 targets had reached 81%, 73%, 59%, respectively, at the end of 2020 [3]. While most countries (Burundi, Eswatini, Kenya, Malawi, Namibia, Lesotho Rwanda, Uganda, Zambia, and Zimbabwe) in the region are close to reaching the 90–90-90 targets, others. like Botswana, have already surpassed them [4]. Most countries now aim at achieving the next target: 95–95-95 [5, 6].

Since 2016, the World Health Organization (WHO) has recommended “Treat All”; ART for all PLHIV, regardless of their clinical or immunological status [7]. A majority of low- and middle-income countries, including those in Sub-Saharan Africa, have adopted this policy: 93% had rolled-out “Treat All” by the end of 2019, while another 2% planned roll-out before the end of 2021 [8]. This recommendation was informed by several clinical trials on “Treat All”, which included universal testing, activities enhancing linkage to HIV care, rapid ART start and patient-centred care [9, 10]. In these trial settings, outcomes improved across the cascade of care, resulting in population-level VL suppression and decreased HIV incidence and mortality. However, despite significant population-level gains, VL suppression was not uniform across all sub-populations, long-term retention in care was not the primary outcome and HIV elimination targets were not reached [9]. Furthermore, there is limited evidence on the real-world effect of “Treat All” across the cascade of care. Clinical trial data are not generalisable to the reality of national programmes, as they are implemented in a controlled environment, often relying on more resources than what is available in routine care. Moreover, patients with a poor clinical condition are often excluded from trial participation [10]. A common term to quantify those not retained on ART is attrition; a combination of death, lost-to-follow-up, and often those who stopped ART and were transferred out [11].

The International Prospective Register of Systematic Reviews (PROSPERO) registered one on-going review assessing strategies to improve linkage to care under “Treat All” [12]. To our knowledge, there are no on-going or published reviews comparing attrition before and after “Treat All” implementation under programmatic conditions in Sub-Saharan Africa. We therefore conducted a systematic review to compare attrition 12 months after ART initiation, before and after the implementation of “Treat All”. We also describe risk factors for attrition as reported by the included studies.

## Methods

We followed the Preferred Reporting Items for Systematic Reviews and Meta-Analyses (PRISMA) guidelines for protocol development and reporting of this review [13, 14]. The protocol was registered on PROSPERO (CRD42020191582) [15]. We used the term before Treat All” for patients who were initiated on ART based on existing guidelines i.e. immunological/clinical criteria or pregnancy/breastfeeding status at a time point prior to the launch of “Treat All”, and we used the term “Treat All” for those who were initiated on ART based solely on an HIV-positive status i.e. at a time point after implementation of the WHO “Treat All” guidelines [7].

### Eligibility criteria

Table 1 shows the components of the search string for population, intervention, comparison outcomes and study designs (PICOs) used for this systematic review.

**Table 1:** Population, intervention, comparison, outcomes, and study design criteria for study inclusion

Criterion	Definition
Population	People living with HIV starting ART under programmatic conditions in Sub-Saharan Africa
Intervention	“Treat All”: ART initiation regardless of clinical or immunological criteria
Comparison	ART initiation according to existing criteria before implementation of “Treat All”
Outcomes	Measures of attrition^a^ at 12 months on ART
Study design	Quantitative retrospective and prospective studies^b^

^a^including attrition, retention

^b^qualitative studies, randomised controlled trials and studies not reporting original patient data (commentaries, viewpoints, letters to editors, reviews, and editorials) were excluded

The WHO “Treat All” recommendation was published in 2016 [7]. We included peer-reviewed studies published since 2018, to allow enough time since the existence of the “Treat All” policy for guideline implementation, collection of programme data and publication. We only included articles which directly compared attrition among PLHIV at 12 months after ART initiation, with data drawn from the same study setting at two different time points, situated before and after “Treat All” implementation in that setting.

### Search strategy

In July 2020, two co-authors (R.M. and T.G.) searched Embase, Google Scholar, Medline through PubMed, and Web of Science for relevant studies with pre-defined search terms (Additional file 1). We searched articles published in the three main academic languages used in Sub-Saharan Africa i.e., English, French and Portuguese. We used the same strings to create alerts in the databases and included additional publications until the end of June 2023. We hand-searched unpublished preprint manuscripts (medRxiv and bioRxiv) and conference abstracts from the following HIV conferences: Conference on Retroviruses and Opportunistic Infections (CROI), International AIDS Society (IAS), International Conference on AIDS and Sexually Transmitted Infections in Africa (ICASA) and International Francophone Conference on HIV, Hepatitis and Sexual Health (AfraVIH) by combining different terms of the PICOs criteria. We also searched for cited references in the articles for which we performed full-text screening.

### Study selection and data extraction

After deduplication, unique results were imported into the Rayyan software and screened independently by two blinded co-authors (R.M. and T.G) in two phases (i.e. titles and abstracts, and full-text screening) [16]. Disagreements between researchers were resolved by discussion and consensus, and when necessary, arbitration by another pair of researchers (co-authors T.D. and L.L.). The two independent researchers (R.M. and T.G.) used a standardised tool for data extraction, collecting information on the study characteristics (e.g., study design, location, period, objectives), participant characteristics (e.g., inclusion/exclusion criteria, baseline characteristics), attrition on ART at 12 months, and predictors of these outcomes as shown in the original publication. We additionally extracted data on pre-ART attrition. When authors only reported outcomes on patients in care (without defining the denominator for ART patients), they were contacted by R.M. to retrieve data restricted to patients on ART.

### Risk of bias assessment

We used a modified Newcastle–Ottawa Scale for cohort studies to assess the risk of bias, based on three parameters (selection, comparability and outcome) [17]. The maximum score a study could get was eight stars (a maximum of four stars was awarded for selection, two for comparability and two for outcome). The results from the Newcastle–Ottawa Scale were classified following the Agency for Healthcare Research and Quality standards of “good”, “fair” and “poor” quality [18].

### Data synthesis

Study and participant characteristics from included studies were tabulated. As most authors reported outcomes as proportions among all PLHIV initiated on ART, we used risk ratios (RRs) to compare attrition at 12 months, before and after “Treat All”. Authors who reported attrition with survival data were contacted to retrieve data restricted to participants with a potential follow-up period of minimum 12 months on ART. Attrition was defined as a composite of death, LTFU and those who stopped ART among all those who initiated ART, including transfers in/out. If only the proportion retained was reported, attrition was defined as 100% minus the proportion retained. A pooled estimate was calculated for attrition, and a forest plot constructed with Stata/IC 16.1 (StataCorp, USA). An inverse variance random-effect model was used for the meta-analysis. Heterogeneity was evaluated using the I-squared statistic, with < 25%, 25–75% and > 75% respectively indicating low, moderate, and high heterogeneity. Predictors of the main outcomes were presented in structured tables.

## Results

### Selection of the included studies

**Fig. 1:**
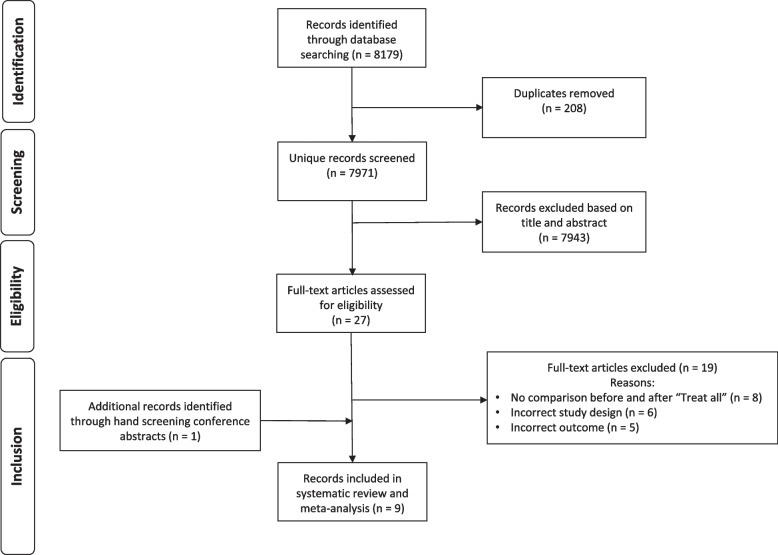
Selection of studies for systematic review to compare attrition 12 months after ART initiation before and after “Treat All” implementation in Sub-Saharan Africa

We identified 8179 records in our databases search and from alerts, and after removing the duplicates, 7971 articles were screened from which 27 [19–46] full-text articles were retrieved for eligibility assessment. From the 27 articles, eight articles [19, 20, 26, 30, 35, 39, 41, 43], and one conference abstract identified through hand search [46] were retained in the systematic review and meta-analysis (Fig. 1).

### Risk of bias assessment for the included studies

The results of the quality assessment are shown in Additional file 2. Of the nine studies, six were scored as “good” [19, 20, 26, 30, 35, 43], one as “fair” [41] and two as “poor” [39, 46]. The study by Awoh et al. was scored poor because no multivariable analysis was conducted [39]. The study by Owona et al. was scored poor because this conference abstract showed limited detail in the methods section [46].

### Characteristics of the studies included

Of the nine studies included, three were from Zimbabwe [20, 26, 41], two from Cameroon [39, 46], one from Kenia [43], one from Malawi [35], one from South Africa [30], and one from the Democratic Republic of Congo [19] (Table 2). Of the nine studies, five included more than 20 health facilities. More than half (5/8) of the studies included both urban and rural facilities, while the others were mainly urban. All the included studies were retrospective cohort studies. Three studies included all patients starting ART [26, 41, 46], three included only adults [20, 30, 39], two included patients above 15 years old [43, 46], and one above 10 years old [35].

**Table 2:** Study characteristics of studies measuring attrition 12 months after ART initiation before and after "Treat All" implementation in Sub-Saharan Africa

Author, year [reference]	Country	Study setting	Study design	Study period (TA implementation)	Study population	Outcome of interest
Alhaj, 2019 [35]	Malawi	32 public clinics (8 urban, 24 rural)	retrospective cohort	BTA: enrolled June 2015, TA: enrolled August 2016 (June 2016)	ART- naïve patients (age > 10 years) starting ART	Retention on ART at 12 months
Awoh, 2019 [39]	Cameroon	Three HIV clinics at tertiary-level hospitals (urban)	retrospective cohort	BTA: enrolled Feb–-April, TA: enrolled July-Sept 2016 (May 2016)	Adult patients starting ART. Transfers in and out excluded	Retention on ART at 12 months
Hirasen, 2020 [30]	South Africa	Two clinics in Johannesburg (urban)	retrospective cohort	BTA: enrolled Dec 2014– May 2015, TA: enrolled Dec 2016– May 2017 (Sept 2016)	ART- naïve adults (age > 18 years) starting ART. Excluded pregnant and TB-infected patients	Outcomes (alive on ART, death, LTFU, viral suppression) at 12 months
Makurumidze, 2020 [26]	Zimbabwe	72 clinics (urban & rural)	retrospective cohort	BTA: April–May 2016, TA: Jan–Feb 2017 (Dec 2016)	Patients starting first-line ART at clinics piloting TA	Retention on ART at 12 months, attrition
Matare, 2020 [41]	Zimbabwe	50 clinics in Harare (urban)	retrospective cohort	BTA: April–June 2015, TA: April–June 2017 (July 2016)	ART-naïve patients starting ART	Retention on ART at 12 months
Mayasi, 2022 [19]	The Democratic Republic of Congo	85 health facilities in Kinshasa (urban)	retrospective cohort	BTA: January 2010–October 2016, TA: Nov 2016–Dec 2019	ART-naïve patients starting ART (age > 15 years)	Retention on ART at 12 months
Mwamuye, 2022 [43]	Kenya	18 HIV clinics (urban & rural)	retrospective cohort	BTA: April − August 2016, TA: April − August 2017 (Sept 2016)	Patients (> 15 years) starting ART	Retention on ART at 12 months
Owona, 2019 [46]	Cameroon	National database (urban & rural)	retrospective cohort (abstract ICASA)	BTA: Oct − June 2016, TA: July 2016–Jan 2017 (June 2016)	Patients starting ART	Retention on ART at 12 months
Tlhajoane, 2021 [20]	Zimbabwe	12 health facilities (predominantly rural)	retrospective cohort	BTA: July 2015–June 2016, TA: July 2016–June 2017 (July 2016)	ART-naïve patients starting ART (age > 18 years)	Attrition on ART at end of follow-up

*BTA* Before "Treat all", *ART* Antiretroviral treatment, *LTFU* Lost-to-follow-up, *TA* "Treat all"

The baseline characteristics (age, sex, WHO stage and CD4 counts) of the study participants for the included studies were summarised by cohort (before or after “Treat All”) (Additional file 3). The median/mean age in years ranged from 31–40 years, and age was similar between before and after “Treat All” cohorts for each of the studies. The proportion of males ranged between 29 and 48% and was similar between the before and after “Treat All” cohorts for the individual studies. The proportion of males was lower compared to females in all studies. In most of the studies, the proportion of patients with stage III/IV was lower in the “Treat All” cohort. Half of the studies reported baseline CD4 counts. The mean CD4 counts (cells/µL) ranged between 194 and 369 before, and between 220 and 308 after “Treat All”, respectively. We contacted Owona et al. to retrieve more information on participant baseline characteristics, but this information was not provided [46].

### Attrition after 12 months on ART before and after “Treat All” implementation

**Fig. 2:**
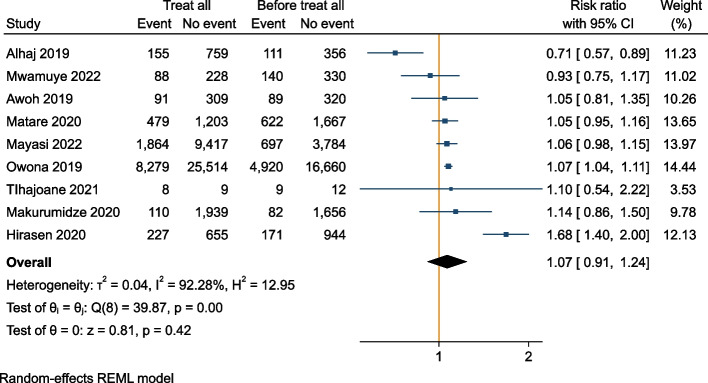
Meta-analysis of attrition 12 months after ART initiation before and after “Treat All” implementation in Sub-Saharan Africa

All studies reported attrition, retention or outcomes from which attrition could be derived 12 months after ART initiation. Three authors who reported attrition by survival analysis were contacted to provide data restricted to those with 12 months potential follow-up on ART. Tlhajoane et al. provided data for a few patients who had 12 months follow-up, while Makurumidze et al. and Mayasi et al. confirmed that none of the included participants was followed for less than 12 months [19, 20, 26]. None of the studies reported pre-ART attrition. Four articles reported a statistically significant higher 12-month attrition after “Treat All” compared to before [19, 26, 30, 46] (Additional file 4). Only one study, by Alhaj et al., reported a significantly higher 12-month retention after “Treat All” compared to before [35]. Data from the nine studies were included in the meta-analysis (Fig. 2).

When study data were reported as RRs, three studies had a statistically significant difference in attrition before and after “Treat All” [30, 35, 46]. The random-effects adjusted pooled RR comparing attrition before and after “Treat All” was not significant [RR = 1.07 (95% CI: 0.91–1.24)] and there was high between-study variation (heterogeneity measured with I^2^ = 92%). Several sensitivity analyses were performed, and the results were generally robust (Additional file 5).

### Predictors of attrition before and after “Treat All” implementation

Five of the included studies investigated predictors of attrition on ART [19, 20, 26, 35, 43] (Table 3). All the studies reported predictors of attrition for the entire study population (including before and after “Treat All”). Four studies used attrition at any time point as an outcome, while Alhaj et al. used attrition 12 months after ART initiation [35]. Patients who started ART under “Treat All” as compared to before had a significantly higher hazard of attrition in two studies, [19, 26] a significantly lower hazard in one study [35], and no significant association was found in two studies [20, 43].

**Table 3:** Predictors of attrition before and after “Treat All” in Sub-Saharan Africa

Author, year [reference]	Outcome	Method of analysis	TA versus BTA	Demographic factors	Stage of HIV disease	Others
Alhaj, 2019 [35]	Attrition at 12 months	Multivariable Cox proportional hazard	aHR: 0.78 [95% CI 0.65–0.92]	Age 20–24 years (vs. 25–49 years)—aHR: 1.53 [95% CI 1.01–2.32] Being pregnant and/or breastfeeding (vs. females) -aHR: 1.87 [95% CI 1.30–2.38] Sex: NS	NR	Facility Urban (vs. rural): NS
Makurumidze, 2020 [26]	Attrition	Multivariable Cox proportional hazard	aHR: 1.73 [95% CI 1.30–2.31]	Male (vs. female)—aHR: 1.45 [95% CI 1.12–1.87] Being pregnant when starting ART (vs not)—aHR: 3.47 [95% CI 2.36–5.11]	WHO stage IV (vs. I–III)—aHR: 2.89 [95% CI 1.16–7.11] Functional status: NS	HIV testing modality, baseline tuberculosis, level of care and partner support: NS
				Confirmed pregancy#TA (versus pregnancy not confirmed#TA)—aHR: 0.48 [95% CI 0.27–0.85]		
				Age: NS		
Mayasi, 2022 [19]	Attrition	Multivariable Cox proportional hazard	aHR: 1.40 [95% CI 1.20–1.50]	Weight—aHR: 0.97 [95% CI 0.96–0.97] Sex, age and pregnancy: NS	Advanced HIV disease—aHR: 1.2 [1.1–1.3]	Days since diagnosis > 7 days (≤ 7 days)—aHR: 0.84 [95% CI 0.74–0.97] Cotrimoxazole prophylaxis (vs. non)—aHR: 0.64 [95% CI 0.57–0.71] Big care centre (vs small)—aHR: 1.3 [95% CI 1.1–1.6] Isoniazid preventive therapy: NS
Mwamuye 2022 [43]	Attrition	Multivariable Cox proportional hazard	aHR: 1.17 [95% CI 0.89–1.54]	Age ≥ 50 years (vs. < 30 years)—aHR: 0.54 [95% CI 0.32 − 0.91] Male (vs. female)—aHR: 1.42 [95% CI 1.07–1.88]	WHO stage, had opportunistic infection: NS	Being divorced/separated (vs. married)—aHR: 1.39 [95% CI 1.01 − 1.92] Nr of adherence sessions missing (vs. ≤ 1)—aHR: 1.79 [95% CI 1.18 − 2.70) Being formally employed (vs. self-employed)—aHR: 0.41 [95% CI 0.22 − 0.74) Being unemployed (vs. self-employed)—aHR: 0.52 [95% CI 0.34 − 0.80)
Tlhajoane, 2021 [20]	Attrition	Competing risk regression	aSHR: 1.12 [95% CI 0.74–1.70]	Sex: NS	Baseline WHO stage, CD4 counts: NS	ART initiation 8–28 days (vs. same day)—aSHR: 0.66 [95% CI 0.43–0.99] ART initiation > 28 days (vs. same day)—aSHR: 0.63 [95% CI 0.40–0.99] Marital status, level of education and health facility characteristics (type, district and level): NS

*ART* antiretroviral therapy, *aSHR* adjusted sub-hazard ratio, *BTA* before “Treat All”, *CI* Confidence interval, *aHR* adjusted hazard ratio, *NR* not reported, *NS* not significant, *TA* “Treat All”, *WHO* World Health Organization

Demographic risk factors for attrition reported both before and after “Treat All” included being an adolescent or young adult, being male and being a pregnant or breastfeeding woman. Two studies reported advanced HIV disease (based on WHO stage III/IV) as a risk factor for attrition. Two studies reported initiation of ART within a week after diagnosis (versus later) as a risk factor for attrition. One study reported that, while pregnancy remained a risk factor for attrition under “Treat All”, the hazard of attrition decreased by 17% after, compared to before, “Treat All” [26].

## Discussion

Our systematic review is the first assessment of potential changes in attrition since the programmatic scale-up of “Treat All” in Sub-Saharan Africa. We found no difference in ART attrition at 12 months before and after “Treat All” in a meta-analysis of nine studies. There was considerable heterogeneity between studies. The reported significant predictors for attrition included advanced HIV, pregnancy and starting ART in the week after diagnosis (versus later).

Our findings on the effect of “Treat All” on attrition are inconclusive. The systematic review did not show a significant difference in attrition at 12 months post ART initiation, when comparing before and after “Treat All” (random effects risk ratio, 1.07, 95% confidence interval: 0.91–1.24). Only nine studies were included in the systematic review. The heterogeneity between studies was very high, in terms of study populations and settings, and study design (including the time between the end of observation of the before “Treat All” and the start of observation of the “Treat All” cohort). The results of the meta-analysis are thus unlikely to reflect the true effect of “Treat All” on attrition. The obtained estimate of the effect of “Treat All” on attrition should therefore be interpreted with caution.

Studies from both low (Cameroun, Democratic Republic of Congo, Kenya) and high (Zimbabwe, South Africa, and Malawi) HIV prevalence settings were included in the systematic review. In low prevalence settings, especially in West-and Central Africa, ART services are often concentrated in urban settings. There is often a delay in adoption of new treatment guidelines in these settings, which can contribute to a poor performance of these programmes along the 90–90-90 HIV care cascades (Cameroon: 78%, 74%, unknown; Democratic Republic of Congo: 75%, 75%, unknown) [1]. Data missing for the 3^rd^ 90 suggest that monitoring of these HIV programmes is suboptimal. On the other hand, high prevalence settings often have well-performing HIV programmes, with better HIV care 90–90-90 cascades (Malawi: 91%, 86%, 81%, South Africa: 92%, 72%, 66% & Zimbabwe: 93%, 93%, 82%) [1]. In Kenya, the well performing ART programme (HIV care 90–90-90 cascade: 96%, 86%, 81%) has even allowed prevalence to drop below the high prevalence threshold of 5% in 2020 [1]. The level of ART coverage before “Treat All” was implemented may also affect outcomes under “Treat All”. Countries with poor performing HIV care cascades, thus with low coverage of ART needs, will have relatively higher proportions of pre-ART patients with advanced HIV disease [19], at risk of attrition. When this group of patients suddenly becomes eligible for ART initiation under “Treat All”, a bulk of patients at risk of attrition will join the after “Treat All” cohort. This may contribute to a higher attrition in the beginning of “Treat All” implementation. In well performing programmes, with a high coverage of ART needs, we speculate that an opposite effect can be observed. Those retained in pre-ART care would have a high CD4 and would be the most motivated patients. The effect of enrolling them on ART under Treat All may have had an opposite effect compared to what happened in poorly functioning programmes. Moreover, the proportion of pregnant and breastfeeding women and TB patients (risk groups known for higher attrition) differed between studies. A study from Malawi, which was at the extreme end in our meta-analysis (attrition higher before “Treat All”), had a higher proportion of pregnant and breastfeeding women in the before “Treat All” cohort as compared to after “Treat All” [35]. A South African study, at the other extreme end (attrition lower before “Treat All”), excluded TB patients and pregnant women, which might have led to proportionally more exclusions from the before “Treat All” compared to the “Treat All” cohort [30]. The differences in the proportion of these known risk groups for higher attrition may help explain the direction of the individual study findings.

The time between the recruitment of two study populations i.e., between before “Treat All” and after “Treat All”, in the included studies was usually short (ranging between 1–9 months). It is thus unlikely that “Treat All” had reached full coverage in all settings when the “Treat All” observation period began. Especially in already malfunctioning programmes/clinics “Treat All” implementation may have been delayed. In settings where implementation was delayed, patients would have remained on pre-ART follow-up, thus would not be included in the “Treat All” cohort. Moreover, the definition of attrition differed significantly between studies. In some studies transfer out was not included in the definition [39] while in others it was included [46].

Despite our efforts to limit the studies included in our systematic review and meta-analysis to those with similar before and after study designs, the included studies used different statistical analysis approaches. Six of the studies presented a cross-sectional analysis of treatment outcomes at 12 months on ART, while the remainder used survival analysis [19, 26, 35]. Survival analysis takes into account time under observation [47]. The difference between the two groups is calculated over the entire period of follow-up (thus not restricted to the first 12 months) and follow-up time differs for each patient. At each point in time, the probability of survival is calculated for those who remained at risk (who have not yet experienced the event) [48]. In our meta-analysis analysis, we transformed survival data to a simple proportion for comparability and to be able to calculate a pooled estimate of attrition before and after “Treat All” at one year after starting ART. We restricted all study populations to those who started ART at least one year before the end of the study period. In the meta-analysis, we used the total number of patients initiated on ART as denominator, and the number with attrition (= dead, LTFU, stopped ART) as numerator. Using either a cross-sectional analysis or a survival analysis may result in different estimates of the effect of “Treat All” on attrition. Calculating individual and pooled estimates based on the number of events and patients at risk at different time points assumes that all follow-up is equal and complete to the relevant time-point. As a result, this method is overly simplistic and possibly unreliable [48]. The studies which used survival analysis and had their data transformed to calculate proportions showed discordant results in the meta-analysis when compared to the results of the survival analysis in the original studies [19, 26, 35]. When the difference between two groups is small, the conclusion may differ, depending on the methodological approach used. Advanced methods to handle survival data in meta-analyses could not be employed due to the smaller sample of studies that had time to event data [47–49].

The before-and-after design, which served as our inclusion criterion, is another reason why the findings of the systematic review and meta-analysis should be interpreted with caution. The primary utility of these studies lies in their ability to show ‘proof of concept’ for an intervention effect [50]. Given the potential for secular changes and other external variables to influence the results between pre- and post- “Treat All” implementation, it is difficult to draw conclusions on the isolated effect of “Treat All” [51, 52]. In reality, many factors independent of “Treat All” could have had an important effect on attrition, such as same day ART initiation, availability of more tolerable ART regimens, better access to monitoring of treatment failure, more decentralisation of care, and growing knowledge on adherence strategies [53, 54]. These factors were not adjusted for in our analysis.

Another issue that makes comparing before and after “Treat All” cohorts difficult is the difference in characteristics of patients starting ART during the two periods, which was influenced primarily by the different guidelines that were implemented during the two periods. Prior to “Treat All”, patients were started on ART based on clinical and immunological criteria (CD4 counts < 500 cells/µL or WHO Stage III/IV) and those not eligible had ART deferred and were followed-up under pre-ART care. Studies done in the before “Treat All” era showed a high level of attrition during the pre-ART period [55, 56]. Only those patients who survived and were retained in care until they were eligible for ART were finally initiated on ART. Patients retained during pre-ART follow-up and who were started on ART thus included those who were most motivated and had engaged with their health facility for a longer time. This selected group of patients were thus also more likely to be retained in care after starting ART [30, 34]. During “Treat All”, everyone diagnosed with HIV is eligible for ART initiation soon after diagnosis, with only a few exceptions receiving pre-ART care (those being treated for opportunistic infections and not ready for life-long treatment). We were not able to account for these differences in terms of characteristics of the study populations of the two periods. Our analysis did not include pre-ART attrition because this was not reported in any of the included studies. As a result, data on those who did not eventually initiate ART were not available, resulting in an underestimation of attrition prior to “Treat All” if all PLHIV were considered. Indeed, when including all patients in ART care before “Treat All”, Onoya et al. found a much lower attrition after “Treat All” was implemented (aHR 0.7, 95% CI: 0.5 to 1.0) [24].

Despite the differences in the effect of “Treat All” on attrition, predictors for attrition pointed in the same direction. Advanced HIV disease, pregnancy and breastfeeding and earlier ART initiation (within one week after HIV diagnosis, compared to later) were associated with attrition across before and after “Treat All” cohorts. Advanced HIV and rapid ART initiation as risk factors for attrition have been reported elsewhere in the post- “Treat all”- era [57]. One study found a reduction (but not annulation) of pregnancy and breastfeeding as a risk factor for attrition after “Treat All” [26]. Additional significant risk factors for attrition found in at least one study were young age, being male, having a lower weight, being divorced, not having data on adherence, not receiving cotrimoxazole and being treated in a small versus large facility.

Since the scale-up, “Treat All” has already had a huge impact on the control of the HIV epidemic. Some countries in Sub-Saharan Africa, where there is the highest HIV-burden, have already met the 90–90-90 targets [3]. Even if the outcomes of those on ART after “Treat All” would be worse, the absolute numbers of PLHIV who are on ART, retained and virologically suppressed are higher. Under “Treat All”, the denominator includes (almost) all PLHIV tested positive; only those not put on ART for clinical or psychosocial reasons may remain under-reported. Before “Treat All”, those ineligible for ART were not shown in clinical programme data, and rarely reported in studies [58]. However, they were also at risk of dying, or likely to be unsuppressed thus transmitting the virus. This translates to increased clinical benefits for a larger number of individuals and reduced transmission overall, which benefits society as a whole.

While “Treat All” should be implemented for both individual and societal benefits, still, potential individual harms in terms of attrition and VL suppression should be mitigated. Integration of ART services (with antenatal care, postnatal care, family planning, immunisation and growth monitoring), use of lay community health workers, such as mothers who have successfully passed through the prevention of mother-to-child programme, and family-centred approaches, should be considered as strategies to improve retention and virologic outcomes in pregnant and breastfeeding women [59]. The advanced HIV care package, which has shown to reduce mortality and morbidity among patients with advanced HIV disease, should be scaled up in low-resource settings [60, 61]. Youth-friendly services, flexible scheduling that considers schooling, and availability of peer caregivers have shown to be effective in retaining adolescents and young adults in care [62, 63]. For men, strategies such as flexible clinic hours to accommodate work and male-friendly services should be considered [64, 65]. However, the identified at-risk groups offer an opportunity to target interventions in resource-restricted settings where it may not be feasible to provide them at scale. Most of the above-mentioned interventions would improve services for everyone (e.g., flexible clinic hours would benefit women as well as men).

Our study has several limitations. Only nine studies were eligible for meta-analysis of attrition at 12 months ART. Because of the small study sample, we did not perform subgroup analysis, as reducing the sample size even more would limit generalisability of the results further. We used attrition after 12 months on ART as an outcome, which might be a short period to measure the true difference in attrition between before and after “Treat All”. All the studies were observational studies, prone to selection and measurement bias. None of the studies reported pre-ART attrition, which is particularly relevant before “Treat All”. Hence, we could not compare outcomes between participants with similar clinical and immunological profiles before and after “Treat All”. We included studies that used a temporal before and after design, i.e., that did measure outcomes in the same study sites at another period. The design has the known limitation of not accounting for the potential for secular changes and other external variables that can influence outcomes. Hence, it was difficult to draw definitive conclusions about Treat All's isolated effect. The differences in outcomes between original articles using survival data and the results of the meta-analysis with RRs shows the importance of the choice of analysis method, and a limitation of meta-analysis using aggregate data. For this meta-analysis, however, it is unlikely that time-adjusted data would have changed the results towards a higher attrition under “Treat All”, since the opposite result was found in time-adjusted analysis by Alhaj et al. [36].

Our study also has some strengths. The studies included cover different geographical regions of Sub-Saharan Africa, and low- and middle-income countries. We employed a rigorous review approach, with two reviewers performing article selection, extraction, and quality assessment in parallel, and a pair of researchers addressing areas of disagreement. We created alerts to get updates on recent articles which were published after our initial search and included yet unpublished conference abstracts in the screening process to address publication bias.

To better understand the effect of attrition on “Treat All” under programmatic conditions, long term implementation data are required. Efforts should be made to conduct future high-quality observational studies with well-defined and measured outcomes and sufficient follow-up time. Since patients starting ART under “Treat All” are not a homogeneous group, more studies should be conducted to assess how each of the subgroups is faring on outcomes. Our review was quantitative, and a qualitative review might assist to understand contextual differences and reasons for differences in attrition.

## Conclusion

In our study, we found the effect of “Treat All” on attrition inconclusive. Considering the benefits of ART on individual health and its impact on HIV transmission, programmes should continue to invest in “Treat All” and optimise its implementation to sustain patient outcomes. Differentiated approaches to enhance retention should be prioritized for those subgroups at risk of poor outcomes.

### Supplementary Information

**Additional file 1.** Search strategy (PubMed) for the systematic review to compare retention and viral suppression before and after HIV “Treat All” implementation in Sub-Saharan Africa

**Additional file 2.** Results Newcastle-Ottawa Scale assessment for cohort studies.

**Additional file 3.** Baseline characteristics of participants in studies measuring retention at 12 months before and after "Treat All" in Sub-Saharan Africa

**Additional file 4.** Reported attrition at 12 months for patients initiating ART before and after "Treat All" implementation in Sub-Saharan Africa

**Additional file 5.** Sensitivity analysis for meta-analysis on attrition 12 months after ART initiation before and after "Treat All" in Sub-Saharan Africa

## Data Availability

All the data which were used in the preparation of this systematic review are part of this publication. Any additional data requests can be sought from Dr Richard Makurumidze, University of Zimbabwe, Faculty of Medicine and Health Sciences. Email: rmakurumidze@medsch.uz.ac.zw.

## Abbreviations

HIV: Human Immunodeficiency Virus
AIDS: Acquired Immunodeficiency Syndrome
ART: Antiretroviral therapy
PLHIV: People living with HIV
WHO: World Health Organization
UNAIDS: Joint United Nations Programme on HIV/AIDS
VL: Viral load
LTFU: Lost to follow up
BTA: Before "Treat All"
TA: "Treat All"
CI: Confidence interval
aHR: Adjusted hazard ratio
RR: Risk ratio
