# Supplementary material for: Attrition one year after starting antiretroviral therapy before and after the programmatic implementation of HIV “Treat All” in Sub-Saharan Africa: a systematic review and meta-analysis
Source: BMC Infect Dis. 2023 Aug 28;23:558. doi: 10.1186/s12879-023-08551-y (PMC10463759; doi:10.1186/s12879-023-08551-y)
Supplement: Supplementary file 1 — Additional file 1. Search strategy (PubMed) for the systematic review to compare retention and viral suppression before and after HIV “Treat All” implementation in Sub-Saharan Africa [file 12879_2023_8551_MOESM1_ESM.docx]

**Additional File**

**Attrition one year after starting antiretroviral therapy before and after the programmatic implementation of HIV “Treat All” in Sub-Saharan Africa: a systematic review and meta-analysis**

Richard Makurumidze ^1, 2, 3*^, Tom Decroo ^1, 4^, Bart K. M. Jacobs ^1^, Simbarashe Rusakaniko ^2^, Wim Van Damme ^1, 3^, Lutgarde Lynen ^1^, Tinne Gils ^1^

**Additional File 1: Search strategy (PubMed) for the systematic review to compare retention and viral suppression before and after HIV “Treat All” implementation in Sub-Saharan Africa**

| **Term subject** | **Terms used** |
| --- | --- |
| **#1 HIV** | ((hiv infections[MeSH Terms]) OR "HIV" OR "AIDS" OR (acquired immunodeficiency syndrome[MeSH terms])) |
| **#2 “Treat All”** | ("Universal testing and treatment" OR "UTT" OR "Test and treat" OR "Treat All" OR "universal treatment" OR "treatment as prevention" OR "TaSP") |
| **#3 ART** | ((Antiretroviral Therapy, Highly Active[Mesh]) OR (Anti-HIV Agents[Mesh]) OR antiretroviral OR anti-retroviral OR antiviral OR anti-HIV OR HAART OR ARV OR ART) |
| **#4 SSA** | ((Africa[Mesh]) OR African OR (Africa South of the Sahara[Mesh]) OR "low resource setting" OR "low-income country" OR Angola OR Benin OR Botswana OR Burkina Faso OR Burundi OR Cameroon OR Canary Islands OR Cape Verde OR Central African Republic OR CAR OR Chad OR Comoros OR Congo OR Democratic Republic of Congo OR DRC OR Djibouti OR Egypt OR Eritrea OR Ethiopia OR Eswatini OR Gabon OR Gambia OR Ghana OR Guinea OR Ivory Coast OR "Cote d'Ivoire" OR Jamahiriya OR Kenya OR Lesotho OR Liberia OR Madagascar OR Malawi OR Mali OR Mauritania OR Mauritius OR Mayotte OR Morocco OR Mozambique OR Namibia OR Niger OR Nigeria OR Principe OR Reunion OR Rwanda OR "Sao Tome" OR Senegal OR Seychelles OR "Sierra Leone" OR Somalia OR St Helena OR Sudan OR "South-Africa" OR "South Africa" OR "Southern Africa" OR "Central Africa" OR Swaziland OR Tanzania OR Togo OR Uganda OR "Western Sahara" OR Zaire OR Zambia OR Zimbabwe) |
| **#5 Outcomes** | (("Treatment Outcome"[Mesh]) OR "lost-to-follow-up" OR "retention" OR "retention in care" OR "attrition" OR "Lost to follow up" OR "lost to follow" OR "lost to follow-up" OR "LTFU" OR "LTF" OR "viral suppression" OR "virologic suppression" OR "virological suppression" OR "viral non-suppression" OR "viral failure" OR "treatment failure" OR "virological failure" OR "high viral load" OR "suppressed viral load" OR "virologic failure" OR "hazard ratio" OR "odds ratio" OR "relative risk") |
| **# 6 (#2 “Treat All” OR #3 ART)** | (((Antiretroviral Therapy, Highly Active[Mesh]) OR (Anti-HIV Agents[Mesh]) OR antiretroviral OR anti-retroviral OR antiviral OR anti-HIV OR HAART OR ARV OR ART)) OR (("Universal testing and treatment" OR "UTT" OR "Test and treat" OR "Treat All" OR "universal treatment" OR "treatment as prevention" OR "TaSP")) |
| **Search terms Final**  #1 HIV AND (#6: (#2 “Treat All” OR #3 ART)) AND #4 SSA AND #5 Outcomes | ((((((Antiretroviral Therapy, Highly Active[Mesh]) OR (Anti-HIV Agents[Mesh]) OR antiretroviral OR anti-retroviral OR antiviral OR anti-HIV OR HAART OR ARV OR ART)) OR (("Universal testing and treatment" OR "UTT" OR "Test and treat" OR "Treat All" OR "universal treatment" OR "treatment as prevention" OR "TaSP"))) AND ((("Treatment Outcome"[Mesh]) OR "lost-to-follow-up" OR "retention" OR "retention in care" OR "attrition" OR "Lost to follow up" OR "lost to follow" OR "lost to follow-up" OR "LTFU" OR "LTF" OR "viral suppression" OR "virologic suppression" OR "virological suppression" OR "viral non-suppression" OR "viral failure" OR "treatment failure" OR "virological failure" OR "high viral load" OR "suppressed viral load" OR "virologic failure" OR "hazard ratio" OR "odds ratio" OR "relative risk"))) AND (((Africa[Mesh]) OR African OR (Africa South of the Sahara[Mesh]) OR "low resource setting" OR "low-income country" OR Angola OR Benin OR Botswana OR Burkina Faso OR Burundi OR Cameroon OR Canary Islands OR Cape Verde OR Central African Republic OR CAR OR Chad OR Comoros OR Congo OR Democratic Republic of Congo OR DRC OR Djibouti OR Egypt OR Eritrea OR Ethiopia OR Eswatini OR Gabon OR Gambia OR Ghana OR Guinea OR Ivory Coast OR "Cote d'Ivoire" OR Jamahiriya OR Kenya OR Lesotho OR Liberia OR Madagascar OR Malawi OR Mali OR Mauritania OR Mauritius OR Mayotte OR Morocco OR Mozambique OR Namibia OR Niger OR Nigeria OR Principe OR Reunion OR Rwanda OR "Sao Tome" OR Senegal OR Seychelles OR "Sierra Leone" OR Somalia OR St Helena OR Sudan OR "South-Africa" OR "South Africa" OR "Southern Africa" OR "Central Africa" OR Swaziland OR Tanzania OR Togo OR Uganda OR "Western Sahara" OR Zaire OR Zambia OR Zimbabwe))) AND (((hiv infections[MeSH Terms]) OR "HIV" OR "AIDS" OR (acquired immunodeficiency syndrome[MeSH terms]))) |
| **+** **Filter for 2018** |  |

**Additional files**

Additional File 1: Search strategy (PubMed) for the systematic review to compare retention and viral suppression before and after HIV “Treat All” implementation in Sub-Saharan Africa

Additional File 2: Results Newcastle-Ottawa Scale assessment for cohort studies.

Additional File 3: Baseline characteristics of participants in studies measuring retention at 12 months before and after "Treat All" in Sub-Saharan Africa

Additional File 4: Reported attrition at 12 months for patients initiating ART before and after "Treat All" implementation in Sub-Saharan Africa

Additional File 5: Sensitivity analysis for meta-analysis on attrition 12 months after ART initiation before and after "Treat All" in Sub-Saharan Africa
