# Supplementary material for: Attrition one year after starting antiretroviral therapy before and after the programmatic implementation of HIV “Treat All” in Sub-Saharan Africa: a systematic review and meta-analysis
Source: BMC Infect Dis. 2023 Aug 28;23:558. doi: 10.1186/s12879-023-08551-y (PMC10463759; doi:10.1186/s12879-023-08551-y)
Supplement: Supplementary file 2 — Additional file 2. Results Newcastle-Ottawa Scale assessment for cohort studies. [file 12879_2023_8551_MOESM2_ESM.docx]

**Additional File**

**Attrition one year after starting antiretroviral therapy before and after the programmatic implementation of HIV “Treat All” in Sub-Saharan Africa: a systematic review and meta-analysis**

Richard Makurumidze ^1, 2, 3*^, Tom Decroo ^1, 4^, Bart K. M. Jacobs ^1^, Simbarashe Rusakaniko ^2^, Wim Van Damme ^1, 3^, Lutgarde Lynen ^1^, Tinne Gils ^1^

**Additional File 2: Results Newcastle-Ottawa Scale assessment for cohort studies.**

|  | **Selection** | | | | **Comparability** | **Outcome** | |  |  |
| --- | --- | --- | --- | --- | --- | --- | --- | --- | --- |
|  | **I** | **II** | **III** | **IV** | **V** | **VI** | **VII** | **Total** | **AHQR standard** |
| Alhaj, 2019 | * |  | * | * | ** | * | * | ******* | Good |
| Awoh, 2019 |  | * | * | * |  | * | * | ***** | Poor |
| Hirasen, 2020 |  | * | * | * | ** | * | * | ******* | Good |
| Makurumidze, 2020 |  | * | * | * | ** | * | * | ******* | Good |
| Matare, 2020 |  | * | * | * | * | * | * | ****** | Fair |
| Mayasi, 2022 | * | * | * | * | ** | * | * | ******** | Good |
| Mwamuye, 2022 | * | * | * | * | ** | * | * | ******** | Good |
| Owona, 2019 (abstract) | * | * | * | * |  | * | * | ****** | Poor |
| Tlhajoane, 2021 |  | * | * | * | ** | * |  | ****** | Good |
|  |  |  |  |  |  |  |  |  |  |
|  |  |  |  |  |  |  |  |  |  |
| **Selection criteria:** |  |  |  |  |  |  |  |  |  |
| I. Representativeness of exposed cohort (⋆: (somewhat) representative) | | | | | |  |  |  |  |
| II. Selection of non-exposed cohort (⋆: drawn from the same community as the exposed cohort) | | | | | | | |  |  |
| III. Ascertainment of exposure (⋆: secure record) | | | |  |  |  |  |  |  |
| IV. Demonstration that outcome of interest was not present at start of study (⋆) | | | | | | | |  |  |
| V. Comparability of cohorts on the basis of the design or analysis (⋆: controls for sex, age, disease progression ⋆⋆: controls also for other factors) | | | | | | | | | |
| VI. Assessment of outcome (⋆: independent blind assessment /record linkage) | | | | | |  |  |  |  |
| VII. Follow-up (⋆: minimum one year, ⋆: complete follow-up or follow-up unlikely to introduce bias) | | | | | | | | | |
| **Agency for Healthcare Research and Quality (AHQR) standards** | | | |  |  |  |  |  |  |
| Good quality: three or four stars in selection domain AND one or two stars in comparability domain AND two or three stars in outcome/exposure domain. | | | | | | | | | |
| Fair quality: two stars in selection domain AND one or two stars in comparability domain AND two or three stars in outcome/exposure domain. | | | | | | | | |  |
| Poor quality: zero or one star in selection domain OR zero stars in comparability domain OR zero or one star in outcome/exposure domain. | | | | | | | | |  |
|  |  |  |  |  |  |  |  |  |  |
|  |  |  |  |  |  |  |  |  |  |
