# Supplementary material for: Attrition one year after starting antiretroviral therapy before and after the programmatic implementation of HIV “Treat All” in Sub-Saharan Africa: a systematic review and meta-analysis
Source: BMC Infect Dis. 2023 Aug 28;23:558. doi: 10.1186/s12879-023-08551-y (PMC10463759; doi:10.1186/s12879-023-08551-y)
Supplement: Supplementary file 3 — Additional file 3. Baseline characteristics of participants in studies measuring retention at 12 months before and after "Treat All" in Sub-Saharan Africa [file 12879_2023_8551_MOESM3_ESM.docx]

**Additional File**

**Attrition one year after starting antiretroviral therapy before and after the programmatic implementation of HIV “Treat All” in Sub-Saharan Africa: a systematic review and meta-analysis**

Richard Makurumidze ^1, 2, 3*^, Tom Decroo ^1, 4^, Bart K. M. Jacobs ^1^, Simbarashe Rusakaniko ^2^, Wim Van Damme ^1, 3^, Lutgarde Lynen ^1^, Tinne Gils ^1^

**Additional File 3: Baseline characteristics of participants in studies measuring retention at 12 months before and after "Treat All" in Sub-Saharan Africa**

| **Study** | | **Cohort** | **Sample size** | **Age** | | **Sex** | **WHO Stage** | **CD4 Counts** | | |
| --- | --- | --- | --- | --- | --- | --- | --- | --- | --- | --- |
|  |  |  |  | **Median or mean** | **IQR or SD** | **Male (%)** | **III—IV (%)** | **Median** | **IQR** | **< 200  cells/µL (%)** |
| Alhaj, 2019 | | BTA | 501 | 33 | 14* | 34 | 26 | ₋ | ₋ | ₋ |
|  | | TA | 991 | 34 | 15* | 39 | 11 | ₋ | ₋ | ₋ |
| Awoh,2019 | | BTA | 423 | 40 | 11* | 34 | ₋ | 194 | 83–367 | ₋ |
|  | | TA | 423 | 40 | 11* | 35 | ₋ | 225 | 101–411 | ₋ |
| Hirasen, 2020 | | BTA | 1267 | 37 | 31–44 | 40 | 24 | 199 | 85–325 | 50 |
|  | | TA^¶^ | 1143 | - | - | 41 | 21 | - | - | 41 |
| Makurumidze, 2020 | | BTA | 1738 | 37 | 30–44 | 36 | 29 | ₋ | ₋ | ₋ |
|  | | TA | 2049 | 36 | 29–43 | 39 | 18 | ₋ | ₋ | ₋ |
| Matare, 2020 | | BTA | 2289 | 31 | 27–41 | 41 | 44 | ₋ | ₋ | ₋ |
|  | | TA | 1682 | 31 | 27–41 | 42 | 27 | ₋ | ₋ | ₋ |
| Mayasi, 2022 | | BTA | 4481 | 39 | 11* | 29 | 54 | 243 | 133–381 | 39 |
|  | | TA | 11281 | 40 | 12* | 34 | 26 | 296 | 187–483 | 27 |
| Mwamuye, 2022 | | BTA | 470 | 39 | 33−48 | 30 | 9.4 | 369 | 173−558 | ₋ |
|  | | TA | 316 | 39 | 32−46 | 33 | 6.9 | 308 | 172−440 | ₋ |
| Owona, 2019 | | BTA | 21590 | ₋ | ₋ | ₋ | ₋ | ₋ | ₋ | ₋ |
|  | | TA | 33793 | ₋ | ₋ | ₋ | ₋ | ₋ | ₋ | ₋ |
| Tlhajoane, 2021^¥^ | | BTA | 254 | 38 | 12 | 41 | 48 | 230 | ₋ | 34 |
|  | | TA | 575 | 37 | 11 | 48 | 22 | 220 | ₋ | 8 |
|  | |  |  |  |  |  |  |  |  |  |
|  |  |  |  |  |  |  |  |  |  |  |
| BTA: Before “Treat Al”, IQR: Interquartile range, -: Not reported, SD: Standard deviation, TA: “Treat All”, WHO: World Health Organization | | | | | | | | | |  |
| * Standard deviation | | | |  |  |  |  |  |  |  |
| ^¶^ Characteristics of patients were stratified by CD4 counts at initiation and medians for whole TA cohort could not be calculated | | | | | | | | | |  |
| ^¥^ The reported baseline characteristics are for whole study though only part of the data were used | | | | | | |  |  |  |  |
|  |  |  |  |  |  |  |  |  |  |  |
