# Supplementary material for: Attrition one year after starting antiretroviral therapy before and after the programmatic implementation of HIV “Treat All” in Sub-Saharan Africa: a systematic review and meta-analysis
Source: BMC Infect Dis. 2023 Aug 28;23:558. doi: 10.1186/s12879-023-08551-y (PMC10463759; doi:10.1186/s12879-023-08551-y)
Supplement: Supplementary file 4 — Additional file 4. Reported attrition at 12 months for patients initiating ART before and after "Treat All" implementation in Sub-Saharan Africa [file 12879_2023_8551_MOESM4_ESM.docx]

**Additional File**

**Attrition one year after starting antiretroviral therapy before and after the programmatic implementation of HIV “Treat All” in Sub-Saharan Africa: a systematic review and meta-analysis**

Richard Makurumidze ^1, 2, 3*^, Tom Decroo ^1, 4^, Bart K. M. Jacobs ^1^, Simbarashe Rusakaniko ^2^, Wim Van Damme ^1, 3^, Lutgarde Lynen ^1^, Tinne Gils ^1^

**Additional File 4: Reported attrition at 12 months for patients initiating ART before and after "Treat All" implementation in Sub-Saharan Africa**

| Author, year | Before “Treat All” | | | After “Treat All” | |  |
| --- | --- | --- | --- | --- | --- | --- |
|  | **Attrition% (95% CI)** | |  | **Attrition% (95% CI)** | |  |
| Attrition lower after “Treat All”  a) Statistically significant difference |  |  |  |  |  |  |
| Alhaj, 2019 | 23.7% | [20.0–27.9] | | 16.9% | [14.5–19.5] | |
|  |  |  | |  |  | |
| b) Statistically insignificant difference |  |  | |  |  | |
| Mwamuye, 2022 | 29.8% | [25.7−34.1] | | 27.8% | [23.0−33.1] | |
|  |  |  |  |  |  |  |
| Attrition higher after “Treat All” |  |  |  |  |  |  |
| a) Statistically significant difference |  |  |  |  |  |  |
| Mayasi, 2022 | 16.0% | [15.0–17.0] | | 17.0% | [16.0–18.0] | |
| Owona, 2021 | 22.7% | [22.2–23.4] | | 24.5% | [24.0–25.0] | |
| Hirasen, 2020 | 15.3% | [13.2–17.5] | | 25.7% | [22.8–28.8] | |
| Makurumidze, 2020 | 4.9% | [4.0–6.0] | | 6.0% | [5.0–7.2] | |
|  |  |  |  |  |  |  |
| b) Statistically insignificant difference | |  |  |  |  |  |
| Matare, 2020 | 27.2% | [25.4–29.0] | | 28.4% | [26.3–30.7] | |
| Tlhajoane, 2021 | 42.8% | [21.8–66.0] | | 47.1% | [23.0–72.2] | |
| Awoh, 2019 | 21.7% | [17.8–26.1] | | 22.7% | [18.7–27.2] | |
|  |  |  |  |  |  |  |
