# Supplementary material for: Attrition one year after starting antiretroviral therapy before and after the programmatic implementation of HIV “Treat All” in Sub-Saharan Africa: a systematic review and meta-analysis
Source: BMC Infect Dis. 2023 Aug 28;23:558. doi: 10.1186/s12879-023-08551-y (PMC10463759; doi:10.1186/s12879-023-08551-y)
Supplement: Supplementary file 5 — Additional file 5. Sensitivity analysis for meta-analysis on attrition 12 months after ART initiation before and after "Treat All" in Sub-Saharan Africa [file 12879_2023_8551_MOESM5_ESM.docx]

**Additional File**

**Attrition one year after starting antiretroviral therapy before and after the programmatic implementation of HIV “Treat All” in Sub-Saharan Africa: a systematic review and meta-analysis**

Richard Makurumidze ^1, 2, 3*^, Tom Decroo ^1, 4^, Bart K. M. Jacobs ^1^, Simbarashe Rusakaniko ^2^, Wim Van Damme ^1, 3^, Lutgarde Lynen ^1^, Tinne Gils ^1^

**Additional File 5: Sensitivity analysis for** **meta-analysis on attrition 12 months after ART initiation before and after "Treat All" in Sub-Saharan Africa**

| **Sensitivity analysis restricted to** | **Studies excluded** | **Risk ratio [95% CI]** | **I2** | **p-value** |
| --- | --- | --- | --- | --- |
| Peer-reviewed studies | Owona et al. | 1.06 [0.89 - 1.27] | 88% | 0.50 |
| Studies including TB patients | Hirasen et al. | 1.01 [0.93-1.11] | 72% | 0.78 |
| Studies including transfers out | Awoh et al. | 1.07 [0.90 -1.27] | 94% | 0.46 |
| Studies rated good or fair quality | Owona et al. & Awoh et al. | 1.07 [0.87 - 1.31] | 90% | 0.55 |
| Studies rated good quality | Owona et al. & Awoh et al. & Matare et al. | 1.07 [0.83 - 1.37] | 89% | 0.60 |
| Studies in which definition of attrition was specified as death and LTFU* | Owona et al. & Awoh et al. excluded | 1.07 [0.87 - 1.31] | 90% | 0.55 |
|  |  |  |  |  |
| **Odds ratio as outcome measure** |  | **Odds Ratio [95% CI]** |  |  |
| All studies | NA | 1.07 [0.91 - 1.24] | 92% | 0.42 |
| * as opposed to 1-retention |  |  |  |  |
